# Central nervous system involvement in chronic inflammatory demyelinating polyradiculoneuropathy—MRS and DTI study

**DOI:** 10.3389/fneur.2024.1301405

**Published:** 2024-01-25

**Authors:** Edyta Dziadkowiak, Magdalena Koszewicz, Przemysław Podgórski, Małgorzata Wieczorek, Sławomir Budrewicz, Anna Zimny

**Affiliations:** ^1^Department of Neurology, Wroclaw Medical University, Borowska, Wrocław, Poland; ^2^Department of General and Interventional Radiology and Neuroradiology, Wroclaw Medical University, Borowska, Wrocław, Poland; ^3^Faculty of Earth Sciences and Environmental Management, University of Wroclaw, Uniwersytecki, Wrocław, Poland

**Keywords:** chronic inflammatory demyelinating polyradiculoneuropathy, diffusion tensor imaging, magnetic resonance spectroscopy, precentral gyrus, sural sensory nerve action potential

## Abstract

**Objective:**

The current research aimed to analyze the alterations within the motor cortex and pyramidal pathways and their association with the degree of damage within the peripheral nerve fibers in patients with chronic inflammatory demyelinating polyradiculoneuropathy (CIDP). To achieve that goal, we investigated the microstructural changes within the pyramidal white matter tracts using diffusion tensor imaging (DTI) parameters, evaluated metabolic alterations in both precentral gyri using magnetic resonance spectroscopy (MRS) ratios, and correlated them with the neurographic findings in patients with CIDP.

**Methods:**

The spectroscopic ratios of NAA/Cr, Cho/Cr, and mI/Cr from both precentral gyri and the values of fractional anisotropy (FA), axial diffusivity (AD), and mean diffusivity (MD) from both of the corticospinal tracts were correlated with the results of neurological and neurographic findings. The comparison of DTI parameters between the patients and controls was performed using Student’s *t*-test or the Mann–Whitney *U* test. Due to the lack of normal distribution of most variables, Spearman’s Rho rank coefficient was used to test all correlations. All analyses were performed at a significant level of alpha = 0.05 using STATISTICA 13.3.

**Results:**

Compared to the control group (CG), the patient group showed significantly lower ratios of NAA/Cr (1.66 ± 0.11 vs. 1.61 ± 0.15; *p* = 0.022), higher ratios of ml/Cr in the right precentral gyrus (0.57 ± 0.15 vs. 0.61 ± 0.08; *p* = 0.005), and higher levels of Cho/Cr within the left precentral gyrus (0.83 ± 0.09 vs. 0.88 ± 0.14, *p* = 0.012). The DTI parameters of MD from the right CST and AD from the right and left CSTs showed a strong positive correlation (0.52–0.53) with the sural sensory nerve action potential (SNAP) latency of the right sural nerve. There were no other significant correlations between other DTI and MRS parameters and neurographic results.

**Significance:**

In our study, significant metabolic alterations were found in the precentral gyri in patients with CIDP without clinical symptoms of central nervous system involvement. The revealed changes reflected neuronal loss or dysfunction, myelin degradation, and increased gliosis. Our results suggest coexisting CNS damage in these patients and may provide a new insight into the still unknown pathomechanism of CIDP.

## 1 Introduction

Typical chronic inflammatory demyelinating polyradiculoneuropathy (CIDP) is a rare, heterogeneous but treatable autoimmune-mediated peripheral neuropathy characterized by demyelination. In this disease, nerve roots and peripheral nerves are damaged. Recent studies have demonstrated that the autonomic involvement in classic CIDP is mild, cholinergic, and mainly sudomotor as a result of lesions occurring at the distal postganglionic axon (1–3). Chronic inflammatory demyelinating polyradiculoneuropathy can occur independently or simultaneously with a variety of diseases, such as monoclonal gammopathy of undetermined significance (MGUS), diabetes, connective tissue disease, and HIV (4–6).

Typical CIDP is more prevalent in men and can occur at any age, with the *highest prevalence* reported in the middle ages (30–60 years of age) (7, 8). The history of the disease is consistently progressive for more than 8 weeks but can be relapsing–remitting (9, 10). The diagnosis is based on clinical suspicion, clinical findings, and the confirmation of demyelinating changes on electrodiagnostic studies (EDX) and nerve pathology (2). Physical examination reveals progressive symmetrical proximal and distal muscle weakness, sensory loss, and decreased or absent deep tendon reflexes. The cranial nerves are less frequently affected (11).

The involvement of the central nervous system (CNS) in patients with CIDP has been reported rarely in the literature. During the past few decades, case reports and small case series have both reported peripheral nervous system (PNS) involvement in multiple sclerosis (MS) and also described CIDP with demyelinating lesions in CNS. For example, in their study on the comparison of CIDP patients with CNS lesions and MS patients with peripheral nervous system involvement, Komori et al. found that 7 of 17 CIDP patients showed CNS involvement (optic neuritis, cerebellar ataxia, and spinal symptoms) and only 2 of 59 MS patients exhibited PNS lesions (12–14). Combined central and peripheral demyelination (CCPD) is a rare condition characterized by heterogeneous features and shows the onset of frequent post-infections, inadequate response to treatments, and generally has a poor outcome. Hypotheses regarding autoimmune mechanisms have been put forward in the pathogenesis of this condition. It is still unclear whether the overlap between central and peripheral demyelination is coincidental or caused by a common epitope in the central and peripheral nervous systems (15–17).

Some authors also pointed out the presence of an association between CIDP and amyotrophic lateral sclerosis (ALS), which both show peripheral nerve demyelination and pyramidal signs with progressive bulbar involvement (18–21).

Novel advanced MR techniques not only enable imaging of the brain structure but also allow for the analysis of brain metabolic and microstructural changes. MR spectroscopy (MRS) provides non-invasive information on the biochemical composition of selected body tissues *in vivo*. The most commonly used MRS technique in clinical practice is hydrogen nuclei spectroscopy (H^1^MRS), which allows the analysis of the profile of brain metabolites, mainly N-acetyl aspartate (NAA), creatine (Cr), choline (Cho), and myoinositol (mI) (22, 23). In particular, the NAA peak is a putative marker of neuronal and axonal integrities, and the choline peak appears to reflect the cell-membrane metabolism. On this basis, a decreased NAA peak is interpreted to represent neuronal/axonal dysfunction or loss, and an elevated choline peak represents increased cell-membrane turnover, as observed in demyelination, remyelination, inflammation, or gliosis. Myoinositol is a part of phospholipids and is only found in astrocytes; thus, it is assumed to be a marker of gliosis (24, 25). Diffusion tensor imaging (DTI) is another advanced MR technique that is used for a detailed evaluation of white matter integrity. It is based on the assessment of the value and direction of water molecule diffusion and the amount of anisotropy within the study structures. There are several mathematical parameters derived from DTI, such as fractional anisotropy (FA), providing information on the direction of water diffusion, mean diffusivity (MD), and axial diffusivity (AD). Fractional anisotropy is the most commonly used DTI metric that seems to reflect the degree of white matter tract packing, myelination, and fiber integrity. On the other hand, MD is an inverse measure of the membrane density and is sensitive to cellularity, edema, and necrosis, while AD is predominantly modified by acute axonal damage (26). DTI is the only method that gives an *in vivo* insight into the microstructure of white matter fibers. The reconstruction of nerve pathways is primarily employed in neurosurgery for surgery planning, where it shows anomalies of the fiber pathways caused by a tumor, with an assessment of the degree of infiltration of white matter tracts or their displacement by pathological brain lesions (27–29). This method has been widely used to assess the integrity of white matter tracts or the rate of early white matter damage in many systemic and brain diseases, such as systemic lupus erythematosus (SLE), MS, Parkinson’s disease (PD), mild cognitive impairment, and Alzheimer’s disease (30–34). What is even more important is that alterations in the parameters of FA or MD could be found even within the normal-appearing white matter.

There is an increasing number of studies that analyze the coexistence of CNS impairment in CIDP. The authors of this study have previously investigated this problem and studied multimodal visual (VEP), auditory brainstem (BAEP), and somatosensory (SEP) evoked potentials (EP) in patients with a diagnosis of CIDP and correlated their results with the electrophysiological parameters of the peripheral sensory nerves. In the previous study, the authors indicated the possibility of central sensory involvement in patients with CIDP, particularly on the basis of prolonged BAEP latencies accompanied by confirmed root damage, and found the degree of central involvement to correlate with the grade of peripheral nerve involvement (35).

The current research aimed to analyze alterations within the motor cortex and pyramidal pathways and their association with the degree of damage within peripheral nerve fibers in patients with CIDP. To achieve that goal, we investigated the microstructural changes within pyramidal white matter tracts using DTI parameters, evaluated the metabolic alterations in both of the precentral gyri using MRS ratios, and correlated them with the neurographic findings in patients with CIDP. To our knowledge, there are no reports in the literature on MRS and DTI cerebral abnormalities in this polyneuropathy.

## 2 Materials

The study included 30 patients (mean age: 57.13 years, range: 23–80 years, 9 women, 21 men) who fulfilled the typical CIDP criteria according to the European Academy of Neurology/Peripheral Nerve Society guidelines on the diagnosis and treatment of chronic inflammatory demyelinating polyradiculoneuropathy from 2021 (8) and 17 age-matched normal control subjects (mean age: 51.9 years, range: 32–79 years, 14 women, 3 men). The Inflammatory Neuropathy Cause and Treatment (INCAT) scale was used to assess the current neurological status, finding a mean upper limb score of 1.40 ± 0.81, a mean lower limb score of 1.53 ± 0.86, and a total mean INCAT of 2.43 ± 1.70. The mean body weight was 82.40 ± 15.83 kg. The co-morbidities showed type 2 diabetes in seven patients and hypertension in eight patients. None of the patients had autoimmune diseases. The laboratory tests revealed the mean level of the following biomarkers: creatine kinase (CK), 407.03 ± 188.56 IU/L; IgG, 10.58 ± 2.62 g/L; IgA, 2.61 ± 0.82 g/L; and IgM, 1.85 ± 0.78 g/L. The general examination of the cerebrospinal fluid (CSF) showed a mean protein level of 66.57 ± 23.22 mg/dL, and a mean pleocytosis amounting to 2.33 ± 1.73 cells/ul. In the patient group, the mean duration of the disease was 4.35 ± 3.20 years. Patients with CIDP were treated with intravenous immunoglobulin, and the duration of treatment was 7.43 ± 11.16 years.

All subjects underwent detailed neurological, biochemical, and electrophysiological examinations (neurography) as well as brain imaging using standard morphological MR sequences followed by MR spectroscopy (MRS) and diffusion tensor imaging (DTI). Subjects with cerebral pathology visible on MRI were excluded from the study.

The exclusion criteria were as follows: patients with known psychiatric or neurological illness other than CIDP, those with any cerebral pathology visible on the structural brain MRI, those who are pregnant, and those who show contraindications for MRI or EMG studies. The inclusion criteria for the healthy control group (CG) included no history of neurological illness or other medical conditions and normal structural brain MRI.

### 2.1 Ethical standards

The study was conducted in accordance with the guidelines of the university ethics committee for conducting research involving humans. Each patient provided signed consent to participate in the examination. The authors had a positive opinion of the Bioethics Committee of the Medical University of Wrocław No. KB—719/2021 on conducting the study. The study was conducted in accordance with the Declaration of Helsinki, with 2013amendments.

## 3 Methods

### 3.1 Electroneurography studies

All patients underwent a subjective and objective neurological examination and serum and CSF analysis. Electrophysiological tests were performed on a Viking Quest version 10.0 device. Standard motor conduction studies were performed in the median, ulnar nerves on the left side and the peroneal, tibial nerves on both sides. The antidromic sensory conduction studies were performed in the left median and ulnar nerves and both sural nerves. In each patient, a particular nerve was examined under the same conditions and at the same distance from the stimulating cathode to the active receiving electrode and at a standardized stimulation site with distal onset latency, amplitude, and conduction velocity assessment. The duration of the electrical stimulation was 0.2 ms for motor fibers and 0.1 ms for sensory fibers. The room temperature was between 21 and 23°C, the hand temperature was not less than 32°C, and the leg temperature was not less than 30°C.

### 3.2 MR studies

All MR studies were performed on a 1.5-T MR scanner (Signa HDx, GE Healthcare, Waukesha, WI, US) using a 16-channel HNS (head–neckspine) coil. The structural MR examination included axial T2-weighted, FLAIR and DWI images as well as coronal and sagittal T2-weighted images followed by 3D T1-weighted structural images acquired using the SPGR 3D BRAVO sequence with the following parameters: TE/TR 5/11 ms, flip angle 13, acquisition matrix 256 × 256 mm2, and FoV 256 × 256 mm.

### 3.3 Magnetic resonance spectroscopy

**Figure 1:**
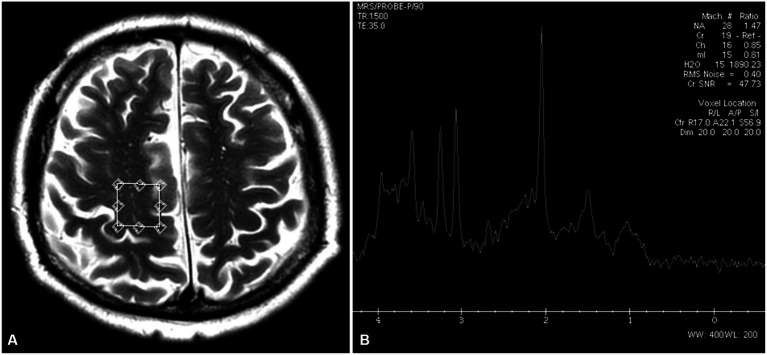
Location of an MRS voxel within the right prefrontal gyrus on a T2-weighted axial image **(A)** and an MRS spectrum with values of NAA/Cr, Cho/Cr, and mI/Cr ratios **(B)**.

H^1^MRS was performed with a single voxel (SV) technique using a point-resolved spectroscopy sequence (PRESS) with the following parameters: TE = 35 ms, TR = 1,500 ms, 128 acquisitions, and NEX 8. In each subject, two 8-cm^3^ (2 × 2 × 2 cm) voxels of interest (VOIs) were prescribed in the right and left precentral gyri anterior to the central sulcus, localized on axial T2-weighted and FLAIR images (Figure 1). The acquisition time for one voxel was 3 min 43 s, and the total time of MRS data acquisition was 7 min 26 s. The MRS data were postprocessed using algorithms provided by a manufacturer (GE workstation, ADW 4.6). Each spectrum was automatically fitted to four peaks corresponding to the levels of NAA (2.02 ppm), total creatine (3.03 ppm), choline-containing compounds (3.23 ppm), and mI (3.56 ppm). The ratios of NAA, choline, and mI to creatine (NAA/Cr, Cho/Cr, mI/Cr, respectively) were calculated and analyzed.

### 3.4 Diffusion tensor imaging

The DTI protocol consisted of a single-shot spin-echo echoplanar imaging sequence with the following parameters: TR/TE 16000/95 ms, FoV 220 × 220 mm^2^, acquisition matrix 90 × 90 mm^2^, reconstruction matrix 90 × 90 mm, and 60 slices with 2.5 mm thickness without gap. Images were performed in the axial plane with diffusion gradients applied in 12 non-collinear directions with a *b*-value of 1,000 s/mm^2^ and one non-diffusion weighted image (36).

The DTI datasets were analyzed using the diffusion MR toolbox “Explore DTI” and consisted of the following steps: (i) correction for subject motion and eddy current-induced distortions (37); (ii) tensor estimation using the REKINDLE approach for outlier detection (38) with iteratively reweighted linear least squares estimation after identification and removal of data outliers (39); and (iii) automated atlas-based analysis within JHU diffusion templates using Diffusion MRI and JHU atlases, registered using affine and elastic registration based on “elastix” (40–49).

**Figure 2:**
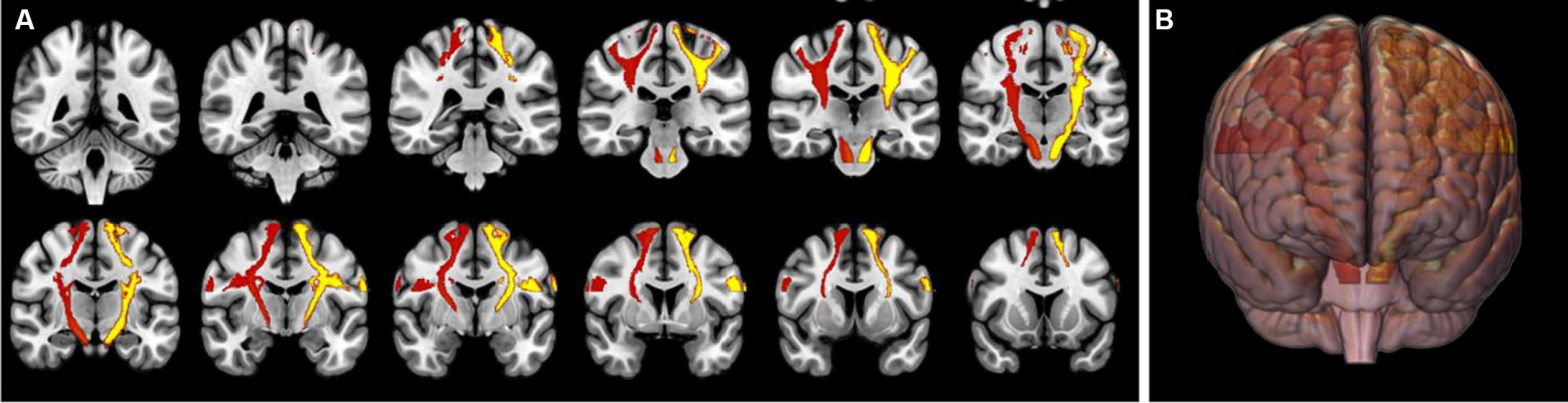
Volume of interests of both of the corticospinal tracts (in red and yellow) at a distance between the precentral gyri and the cerebral peduncles overlaid on a 2D coronal structural T1-weighted image **(A)** and a 3D brain visualization **(B)**.

All DTI data were visually checked in terms of the quality of tensor estimation and the quality of registration. After following all preprocessing steps, the mean values of FA, MD, and AD were calculated based on the JHU atlas separately for the right and left corticospinal tracts at a distance between the precentral cortex and the cerebral peduncle (Figure 2).

### 3.5 Data analysis

Spectroscopic ratios of NAA/Cr, Cho/Cr, and mI/Cr from both precentral gyri as well as the values of FA, AD, and MD from both corticospinal tracts were correlated with the results of neurological and neurographic findings. The analysis was performed separately for the values from the right and left sides.

The comparison of DTI parameters between the patient group and the CG was performed using Student’s t-test (when the subgroups had a normal distribution) or the Mann–Whitney *U* test (when there was no normal distribution). Student’s *t*-test was used when comparing the statistical differences of patients and the control group in terms of MRS.

Due to the lack of normal distribution of most variables, Spearman’s Rho rank coefficient was used to test all correlations. All analyses were performed at a significance level of alpha = 0.05 using STATISTICA 13.3.

## 4 Results

MRS was performed in 30 patients, and the results are shown in Table 1. Compared to the CG, the patient group showed significantly lower ratios of NAA/Cr and higher ratios of ml/Cr in the right precentral gyrus as well as higher levels of Cho/Cr within the left precentral gyrus.

**Table 1:** Results of MRS from the right and left precentral gyri in patients with CIDP and healthy controls (CG).

MRS ratios brain location	CIDP group		Control group		*p*-value
	Mean	SD	Mean	SD	
NAA/Cr R precentral gyrus	1.61	0.15	1.66	0.11	0.022*
Cho/Cr R precentral gyrus	0.85	0.13	0.84	0.09	0.316
mI/Cr R precentral gyrus	0.61	0.08	0.57	0.15	0.005*
NAA/Cr L precentral gyrus	1.59	0.19	1.64	0.14	0.060
Cho/Cr L precentral gyrus	0.88	0.14	0.83	0.09	0.012*
mI/Cr L precentral gyrus	0.66	0.13	0.62	0.05	0.062

NAA, N, acetylaspartate; Cr, creatine; Cho, choline; mI, myoinositol; R, right; L, left; SD, standard deviation; *, statistically significant result.

DTI studies were performed in 20 patients in the patients group and in 17 subjects from the CG. The results are presented in Table 2. There were no significant differences in DTI measurements within both CSTs between the groups.

**Table 2:** DTI results from the corticospinal tracts in patients with CIDP and healthy controls (CG).

DTI parameters brain location	CIDP group		Control group		*p*-value
	Mean value	SD	Mean value	SD	
FA Corticospinal tract R	0.294	0.049	0.318	0.110	0.726
FA Corticospinal tract L	0.313	0.062	0.318	0.094	0.867
MD Corticospinal tract R	0.001	0.0001	0.001	0.0001	0.772
MD Corticospinal tract L	0.001	0.0001	0.001	0.0001	0.788
AD Corticospinal tract R	0.001	0.0001	0.001	0.0001	0.451
AD Corticospinal tract L	0.001	0.0001	0.001	0.0001	0.812

FA, fractional anisotropy; MD, mean diffusivity; AD, axial diffusivity; R, right; L, left; SD, standard deviation.

**Figure 3:**
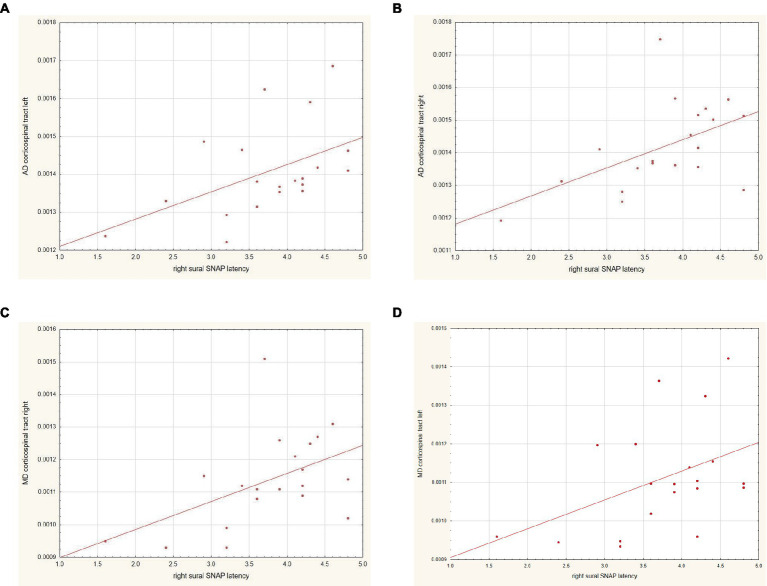
Correlation between the results of **(A)** AD within the left corticospinal tract and SNAP latency within the right sural nerve, **(B)** AD within the right corticospinal tract and SNAP latency within the right sural nerve, **(C)** MD within the right corticospinal tract and SNAP latency within the right sural nerve, and **(D)** MD within the left corticospinal tract and SNAP latency within the right sural nerve.

The DTI parameters of MD from the right CST and AD from the right and left CSTs showed a strong positive correlation (0.52–0.53) with the SNAP latency of the right sural nerve (Figure 3). There were no other significant correlations found between other MRS or DTI parameters and neurographic results. There were no significant correlations between MRS or DTI parameters and disease duration, the severity of the disease, the duration of IgIV treatment, or several biochemical parameters, such as creatine kinase, IgG, IgA, IgM in blood tests, the protein level, and the mean CSF pleocytosis. There were no significant correlations between the DTI and MRS results.

## 5 Discussion

In our study on patients with CIDP, we found significantly lower values of NAA/Cr ratio and higher Cho/Cr and mI/Cr ratios in both precentral gyri, which may indicate a decrease in the number of normal neuronal cells or axons, myelin degradation, and an increase in astroglial proliferation and gliosis in these brain regions. The precentral gyri are the locations of the primary motor cortex (Brodmann’s field 4) that is responsible for the control of voluntary motor movements. The precentral gyrus is also the origin of several motor pathways, such as the corticospinal, corticobulbar, and cortico-rubrospinal tracts. The fibers then intermix with fibers from the lateral corticospinal tract and travel down the spinal cord in the lateral funiculus. The axons from the rubrospinal tract then synapse on alpha and gamma motor neurons of the muscles associated with the movements of the extremities (50). Though CIDP is a disorder of the peripheral nervous system resulting from the deterioration of the myelin sheath, sporadic case reports of combined central and peripheral demyelination (CCPD) have been reported. CCPD is a large term that was proposed to describe a situation associated with demyelinating lesions in both the central and peripheral systems (CNS and PNS) (51, 52). The results of our study prove that, in typical CIDP, there is also brain involvement, and metabolic changes may be detected even in the normal-appearing precentral gyri. To our knowledge, this is the first study to show biochemical abnormalities in these locations in CIDP. Typically, the pathology within both precentral gyri has been associated with ALS, and in this disease, MRS studies were also carried out. Their results show similar changes such as reduced NAA/Cr in the precentral gyrus (53). The coincidence of sensorimotor demyelinating polyneuropathy with ALS is presented in the literature as case reports. The pathomechanism of peripheral nerve demyelination in ALS cannot be explained simply as secondary temporal demyelination to progressive axonal degeneration. Case reports demonstrate neurographic lesions of peripheral nerve sensory fibers, the presence of anti-ganglioside antibodies, and alterations in sural nerve biopsy typical of CIDP (21, 54–56). Echaniz-Laguna et al. presented a postmortem study in one patient with coexisting CIDP and ALS, which showed changes typical of CIDP (among others, mononuclear cell infiltration in the lumbar roots and distal and proximal peripheral nerves) and lesions typical of ALS (among others, loss of axons in the corticospinal tracts, and loss of neurons in the anterior horn) (56). The co-occurrence of CIDP and ALS may lead to the hypothesis that precentral gyrus lesions are involved in the pathomechanism of CIDP, and indeed, in our study, metabolic alterations in this region were found in patients with CIDP without any coexistence of clinical symptoms typical of ALS.

In our study, we did not find any significant alterations in either CSTs using DTI parameters. To our knowledge, there are no other reports on DTI findings from the brain in patients with CIDP. On the other hand, there are several studies on the application of DTI in the assessment of peripheral nerves in conditions of entrapment neuropathy, tumors, and traumatic injury (57) and in the assessment of the rate of damage in CIDP and axonal polyneuropathies (58, 59). DTI and DTT of the peripheral nerves have the potential to introduce novel functional information beyond conventional, qualitative MRI. DTI of proximal nerve segments may be useful for estimating the proximal axonal degeneration burden in patients with peripheral neuropathies (57–59). Wu et al. concluded that the cross-sectional area (CSA) and apparent diffusion coefficient (ADC) values of the lumbosacral nerve roots could help identify patients with CIDP and further distinguish them from patients with axonal polyneuropathies (59).

On the other hand, FA alterations were found in CST in other clinical conditions, especially in ALS. Decreased FA in the CTS is the main DTI finding in ALS. One of the recent studies reported that significantly decreased FA and AD values in the CST were observed only at the level of the brainstem, which could be due to the fact that the tract fibers are tightly packed in this brain location and that the DTI values at the brainstem level could be more sensitive to structural changes (60). The lack of significantly decreased FA values in CST in our study may be partially related to the methodology that was used to estimate DTI parameters within CSTs as averaged values from the whole bundle between the precentral cortex and the cerebral peduncle. In such an approach, subtle disturbance within these tracts could have been averaged and not detected.

In our study, we found no correlations between MRS parameters and the results of neurography within the motor nerves. The authors hypothesize that, although patients with CIDP show metabolic alterations in the precentral cortex and demyelination in the peripheral nervous system, there are no linear associations between these two processes as they may occur independently. A positive correlation was found between the sural nerve SNAP latency and DTI parameters. According to the European Academy of Neurology/Peripheral Nerve Society guidelines on the diagnosis and treatment of CIDP, it is suggested not to perform nerve biopsy as a routine procedure (8). However, Kulkarni et al. showed that histopathological changes from sural nerve biopsy were present in 100% of the examined patients, whereas electrophysiological abnormalities were detected in 90.8% (21) of them, which indicates the importance of the sural nerve damage in the diagnosis of the disease. Thus, the sural nerve may serve as a pathognomonic site of peripheral nervous system injury in CIDP. In this study, we hypothesize that the prominent aggravation of the autoimmune process manifested in sural nerve dysfunction may also be manifested as an impairment to CSTs.

In our study, we found no significant correlations between DTI and MRS parameters and disease duration, severity of the disease, duration of IgIV treatment, or several biochemical studies such as creatine kinase, IgG, IgA, IgM of blood tests, the protein level, and the mean CSF pleocytosis. Damage to the CNS is probably subtle, and we found only changes in the metabolic composition within the precentral gyri. The lack of correlations with the disease duration, disease severity, or other biochemical markers may be due to other mechanisms causing the CNS damage and due to the fact that this process may not be directly associated with the damage to the peripheral nervous system and may occur independently and with different characteristics.

### 5.1 Limitations

Our study has several limitations. One of them is a relatively small group of patients with CIDP. Another one is the method of evaluation of CTS using DTI parameters as the mean values from the whole pyramidal tract length between the precentral gyrus and the cerebral peduncle, which may not be able to detect subtle microstructural alterations and thus could be the reason for the lack of significant changes between the CIDP patients and the CG.

## 6 Conclusion

In our study, significant metabolic alterations were found in the precentral gyri in patients with CIDP without clinical symptoms of CNS involvement. The revealed changes reflected neuronal loss or dysfunction, myelin degradation, and increased gliosis. Our results suggest coexisting CNS damage in these patients and may give a new insight into the still unknown pathomechanism of CIDP. A better understanding of the whole spectrum of changes in CIDP may benefit in the improvement of treatment strategies and therapies in the future.

## Data availability statement

The raw data supporting the conclusions of this article will be made available by the authors, without undue reservation.

## Ethics statement

The studies involving humans were approved by the Bioethics Committee of the Medical University of Wrocław (No. KB‐719/2021). The studies were conducted in accordance with the local legislation and institutional requirements. The participants provided their written informed consent to participate in this study.

## Author contributions

ED: Data curation, Formal analysis, Writing – review & editing. MK: Formal analysis, Writing – review & editing. PP: Data curation, Formal analysis, Investigation, Writing – review & editing. MW: Formal analysis, Investigation, Writing – review & editing. SB: Data curation, Writing – review & editing. AZ: Data curation, Formal analysis, Writing – review & editing.
